# Protein Adsorption and Its Effects on Electroanalytical Performance of Nanocellulose/Carbon Nanotube Composite Electrodes

**DOI:** 10.1021/acs.biomac.3c00449

**Published:** 2023-07-11

**Authors:** Touko Liljeström, Katri S. Kontturi, Vasuki Durairaj, Niklas Wester, Tekla Tammelin, Tomi Laurila, Jari Koskinen

**Affiliations:** † Department of Chemistry and Materials Science, School of Chemical Technology, Aalto University, P.O. Box 16100, 00076 Aalto, Finland; ‡ Sustainable Products and Materials, VTT Technical Research Centre of Finland, P.O. Box 1000, FI-02044 Espoo, Finland; § Department of Electrical Engineering and Automation, School of Electrical Engineering, Aalto University, P.O. Box 13500, 00076 Aalto, Finland

## Abstract

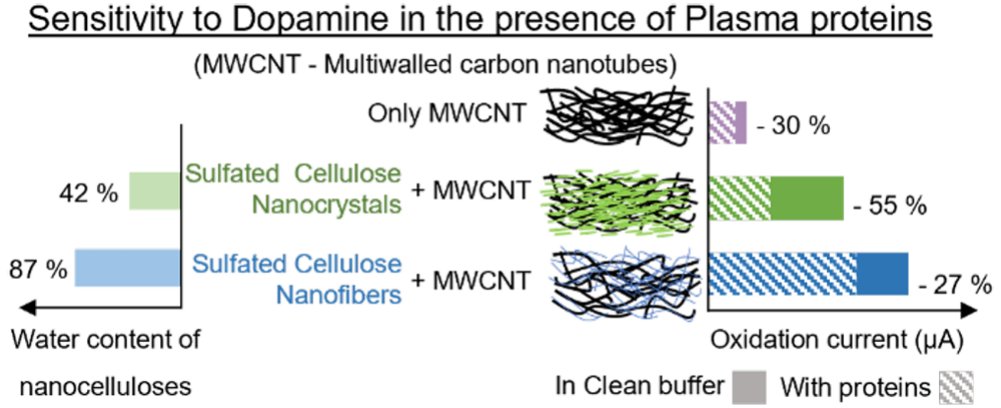

Protein fouling is a critical issue in the development of electrochemical sensors for medical applications, as it can significantly impact their sensitivity, stability, and reliability. Modifying planar electrodes with conductive nanomaterials that possess a high surface area, such as carbon nanotubes (CNTs), has been shown to significantly improve fouling resistance and sensitivity. However, the inherent hydrophobicity of CNTs and their poor dispersibility in solvents pose challenges in optimizing such electrode architectures for maximum sensitivity. Fortunately, nanocellulosic materials offer an efficient and sustainable approach to achieving effective functional and hybrid nanoscale architectures by enabling stable aqueous dispersions of carbon nanomaterials. Additionally, the inherent hygroscopicity and fouling-resistant nature of nanocellulosic materials can provide superior functionalities in such composites. In this study, we evaluate the fouling behavior of two nanocellulose (NC)/multiwalled carbon nanotube (MWCNT) composite electrode systems: one using sulfated cellulose nanofibers and another using sulfated cellulose nanocrystals. We compare these composites to commercial MWCNT electrodes without nanocellulose and analyze their behavior in physiologically relevant fouling environments of varying complexity using common outer- and inner-sphere redox probes. Additionally, we use quartz crystal microgravimetry with dissipation monitoring (QCM-D) to investigate the behavior of amorphous carbon surfaces and nanocellulosic materials in fouling environments. Our results demonstrate that the NC/MWCNT composite electrodes provide significant advantages for measurement reliability, sensitivity, and selectivity over only MWCNT-based electrodes, even in complex physiological monitoring environments such as human plasma.

## 1 Introduction

Electrochemical sensors are a highly promising technology for fast and easy detection of small molecules in the healthcare field,^1^ where reliable and quick monitoring of changes in their concentrations can be vital for patient care.^2^ Conventional carbon-based electrodes such as carbon paste^3^ graphite rod,^4^ carbon fiber,^5^ boron doped diamond^6^ and glassy carbon^7^ electrodes have been used extensively for development of electrochemical sensors owing to some highly attractive properties of carbon such as low cost, high chemical stability, and wide operation potential. However, the sensitivity of bulk carbon electrodes is often limited due to their low surface area, and consequently the use of carbon nanomaterials for surface modifications has become increasingly popular,^8−10^ as they offer larger reactive surface area, low charge transfer resistance, and improved reaction kinetics.^8,11,12^ Tetrahedral amorphous carbon (ta-C) thin films are mechanically robust and chemically inert electrode materials that can be produced using complementary metal–oxide–semiconductor (CMOS) compatible deposition processes.^13,14^ Such ta-C electrodes modified with carbon nanomaterials like partially reduced graphene oxide (PRGO),^15^ nanodiamonds,^16^ and carbon nanotubes^17,18^ exhibit drastically improved performances for the detection of various small molecules. Further, three-dimensional structures with carbon nanomaterials show higher resistance to both electrochemical fouling and biofouling, compared to planar architectures.^19^

Commercial multiwalled carbon nanotubes (MWCNTs) are of special interest for nanostructured surface modifications of electrodes, owing to their relatively low cost and high yield industrial production capabilities. However, their poor dispersibility in both aqueous and nonaqueous media requires various chemical or physical functionalization methods to disperse the MWCNT for developing electrochemical platforms.^20,21^ Chemical functionalization of MWCNT with nitric acid or a mixture of nitric acid and concentrated sulfuric acid is one of the most used techniques, which results in the introduction of a high density of various oxygen functionalities (mainly carboxyl groups) at the tube ends, defect sites and side walls of the CNT.^22^ Several research works developing MWCNT-based electrochemical sensors use this process to obtain carboxylated MWCNT, which are then further chemically or physically modified to improve sensitivity and selectivity for various analytes.^23−25^ Carboxylated MWCNT-modified screen-printed electrodes are also available commercially, for example, from Metrohm DropSens S.L., and are used here in this work as a commercial reference standard. Specialized techniques such as self-assembly or molecular imprinting can result in highly controlled nanoscale architectures of CNT modified electrodes, but their increased economic and environmental costs limit their large-scale industrial production and commercial applications.^26^

Use of nanocellulosic materials for the dispersion of carbon nanomaterials^27^ provides a promising alternative for the sustainable and economic development of electrochemical sensor platforms and has attracted significant attention in recent years.^28−30^ The natural abundance of cellulose, along with rapidly developing processing technologies for nanocellulose (NC) extraction and functionalization offer great potential for their use in several fields.^31,32^ We have recently shown that nanocellulosic materials with varying functionalizations and geometries can be used to successfully develop robust, composite electrochemical platforms with commercial MWCNT,^33,34^ without the need for any additional modifications to the MWCNTs, which may often decrease electrical conductivity or cause deterioration of their electrochemical properties.^35−37^ Further, the inherent hygroscopic nature of NC has been shown to have improved biofouling resistance in several applications.^38,39^ Thus, it is reasonable to expect that the NC composites with MWCNTs would have superior fouling resistance properties, compared to more hydrophobic, purely MWCNT-based architectures. It can also be hypothesized that the nature of the functionalized nanocellulosic component would have a considerable influence on the performance of resultant NC/MWCNT composite in different fouling environments. All nanocellulosic grades are known for their high hydration tendency and water responsiveness.^40,41^ Hence, within this context, two NC grades were selected based on the expectedly stark contrast with respect to their hydration behavior: (i) high charge, flexible sulfated cellulose nanofibers (SCNF) with pronounced water uptake, and (ii) low charge, stiff sulfated cellulose nanocrystals (SCNC) with (relatively) minor water uptake. Composite electrodes were prepared by mixing these nanocellulosic materials with commercial unmodified MWCNT.

Physiological protein fouling effects are often estimated in research using only bovine serum albumin (BSA),^42−45^ but human plasma consists of various coagulation factors, fibrinolytic proteins and immunoglobulin together with multivalent ions in addition to albumin, making it a much more complex environment for electrochemical sensing.^45^ Thus, in the present study, we investigate the protein adsorption mechanisms and their corresponding effects on the electrochemical performance using both BSA and human plasma. Two benchmark cationic molecules, hexaammineruthenium(III) chloride (Ru(NH_3_)_6_Cl_3_, hereafter referred to as RuHex) and dopamine (DA), are chosen in this study as analytes of interest. Cyclic voltammetry (CV) measurements are carried out immediately upon immersion of electrodes and after 5 min of incubation in the different environments to evaluate the effects of electrode fouling. Hydration effects and fouling tendencies of the individual electrode components are also analyzed in the various studied environments using quartz crystal microgravimetry with dissipation monitoring (QCM-D). Our studies indicate that the NC/MWCNT composite electrodes provide significant advantages for measurement reliability, sensitivity, and selectivity over only MWCNT-based electrodes, even in complex physiological monitoring environments such as human plasma.

## 2 Experimental Section

### 2.1 Materials

The functionalized nanocellulosic materials used in this work, sulfated cellulose nanofibers (SCNF) and sulfated cellulose nanocrystals (SCNC), were prepared and characterized using methods described in previous work (Supporting Information Figure S-1).^46,47^ In brief, (i) SCNC was prepared by sulfuric acid hydrolysis of cotton cellulose paper^46^ and (ii) SCNF was prepared by direct sulfation of softwood dissolving cellulose pulp using a deep eutectic solvent mixture of sulfamic acid and urea.^47^ Commercial MWCNT was purchased from NanoLab Inc. (Newton, MA) in the dry form. MWCNT-modified commercial screen-printed electrodes were purchased from Metrohm DropSens S.L., Spain, to be used as a reference standard. Poly(etheleneimine) (PEI) solution (branched polymer), hexaammineruthenium(III) chloride, dopamine hydrochloride, and bovine serum albumin (BSA) were purchased from Sigma-Aldrich. Undiluted human plasma (OctaplasLG) was received from Octapharma AB, Sweden.

### 2.2 Methods

#### 2.2.1 Quartz crystal microgravimetry with dissipation monitoring (QCM-D)

QCM-D was used to characterize the hydration tendencies of the different NC films and to evaluate the interactions of BSA or human plasma proteins with the individual nanocellulosic materials and a model sp^2^-rich carbon surface. Single-component NC model films were prepared for the QCM-D studies by spin-coating ultrathin films of SCNC and SCNF on AT cut gold-covered QCM-D sensors with a fundamental resonance frequency *f*_0_ ≈ 5 MHz (Biolin Scientific, Gothenburg, Sweden), using a thin self-adsorbed layer or polyethylene imine (PEI) as an anchor. Due to the difficulties in obtaining ultrathin uniform films of pure MWCNT suitable for QCM-D, a surface sp^2^-rich tetrahedral amorphous carbon (ta-C) thin film, deposited on silica-coated QCM-D sensor, was used to represent the MWCNT surface, due to the similarities in their sp^2^-hybridized carbon structure.^48^ Detailed QCM-D sample preparation protocols are provided in the Supporting Information. QCM-D measurements were conducted with an E4 Analyzer instrument (Biolin Scientific, Gotherburg, Sweden). To characterize the dry mass of the deposited NC films, the mass of the QCM-D sensors was measured under normal atmospheric conditions before and after the film deposition and subsequent rinsing and drying. Hydration of the NC films under liquid water was quantified by H_2_O/D_2_O solvent exchange method^49^ at 20 °C. Prior to solvent exchange, the cellulose films deposited on the QCM-D sensors were swollen overnight in water, after which the reversible change in resonance frequency, (Δ*f*/*n*)_film_, induced by water-D_2_O exchange, was monitored. Together with the respective response measured for a bare QCM-D sensor (Δ*f*/*n*)_bare_, and the known values for densities of H_2_O and D_2_O (ρ_H_2_O_, ρ_D_2_O_), calculation of contribution of the bound water by the cellulose film on the resonance frequency (Δ*f*/*n*)_H_2_O_ is possible using the equation:
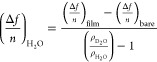
1and the total water content (mg/m^2^) of the film, Γ_H_2_O_ can be calculated according to the Sauerbrey equation:^50,51^
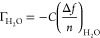
2where *C* is the sensitivity constant of the device (17.7 ng Hz^–1^ cm^–2^ for a 5 MHz crystal). Protein interaction measurements were conducted in 10 mM PBS buffer at pH 7.4. Prior to measurements, the sensors were stabilized overnight in a buffer solution. After stabilization of the baseline, adsorption from 1 g/L BSA solution or 1:39 diluted human plasma solution was followed for ∼1 h, after which the surface was again rinsed with pure buffer solution for ∼1 h. Each solution was passed through the QCM-D cell with a flow rate of 0.1 mL/min. The data presented was acquired using the fifth overtone (25 MHz, *f*_0_ = 5 MHz, *n* = 5). The thicknesses of the hydrated adsorbed protein layers were assessed either with Sauerbrey or viscoelastic modeling of QCM-D data utilizing Dfind software (Biolin Scientific), selected based on the viscoelastic nature (*ΔD* values) of the layer.

#### 2.2.2 Atomic force microscopy (AFM)

AFM images were obtained using an Anasys afm+ instrument (Anasys Instruments Inc., Santa Barbara, CA, USA), to determine the morphology of the ultrathin component films used as the QCM-D samples. The images were scanned in tapping mode in air at 25 °C using ACTA silicon cantilevers with spring constant 37 N/m (Applied Nanostructures Inc., Mountain View, CA, USA). No image processing except flattening was done.

#### 2.2.3 Nanocellulose/MWCNT Suspensions

The NC/MWCNT suspensions were prepared by adding 0.0125 g of as obtained dry MWCNT (no pretreatments used) to either SCNF or SCNC aqueous suspensions (prediluted to 0.25 wt %), resulting in a total suspension weight of 10 g and a dry weight percentage ratio of 2:1 (NC:MWCNT). The suspensions were then tip sonicated for 10 min (Qsonica Q500) in an ice bath using a 2 mm probe at 20 kHz (30 W), in pulsed mode (5 s on, 1 s off). The resulting suspensions were then stored in a refrigerator (ca. 5 °C) and have been found to be stable under visual inspection for over 2 years at the time of writing. The suspension stability was further verified with UV–vis spectroscopy measurements and by comparing the electrochemical responses of the composite electrodes made in years 2020 and 2022 (used in this work) from the same suspensions (details provided in Supporting Information Figures S-2 and S-3).

#### 2.2.4 Electrochemical Measurements

The electrochemical measurements were conducted using a Gamry Reference 600 potentiostat, in a conventional three-electrode setup using a leakless Ag/AgCl reference electrode (+0.199 V vs SHE) from eDAQ and a platinum wire counter electrode. The NC/MWCNT composite modified working electrodes were prepared on 7 nm ta-C thin-film coated Si substrates, using the same protocol described in detail in previous work.^33^ In brief, a boron-doped silicon wafer was sputtered with a 20 nm-thick Ti adhesive layer, followed by pulsed filtered cathodic vacuum arc deposition of a 7 nm ta-C layer. The wafer was diced into 5 mm by 5 mm pieces using a dicing saw. Individual pieces were packaged into electrodes with a 0.07 cm^2^ circular contact area, as has been reported in previous works.^18,33^ The NC/MWCNT composite modification was achieved by first forming a self-adsorbed layer of PEI on the exposed ta-C electrode area, followed by drop-casting 7 μL of the NC/MWCNT suspension and then drying at 80 °C under ambient pressure for 1 h. Commercial screen-printed electrodes from Metrohm DropSens containing carboxylated MWCNTs (DRP110CNT) were used as a reference standard, and for the sake of comparability, the commercial electrodes were covered with Teflon tape such that only a 3 mm diameter circular area of the working electrode was exposed to the measurement solution, and external Ag/AgCl and platinum wire were used as reference and counter electrodes. Two analytes, 1 mM hexaammineruthenium(III) chloride (RuHex) and 100 μM dopamine (DA), were measured in three different electrochemical environments, namely 10 mM phosphate-buffered saline (PBS) solution (pH = 7.4), 4 wt % BSA diluted in PBS (2 g in 50 mL), and human blood plasma. The final dilution of human plasma while adding different analytes was kept <10% in all measurements. Before electrochemical measurements, all electrodes were kept immersed in PBS electrolyte solution for about 45 min to achieve stable wetting and swelling of the NC matrix. Between different measurements, the electrodes were returned to the beaker with a blank PBS solution and kept immersed. Cyclic voltammetry (CV) measurements were used for evaluating the electrochemical behavior. All electrodes were subjected to background cycling in 10 mM PBS in a wide potential window (−0.6 to 0.9 V) first for 25 cycles, to establish a stable electrochemical background. Electrode behaviors in different fouling environments were investigated over a 5 min incubation period, where after the introduction of the electrode to the measurement solution, an immediate measurement cycle was run with a scan rate of 100 mV/s. This was followed by a 5 min incubation period, during which the electrode was kept in the measurement solution, after which another 100 mV/s scan rate cycle was run. Dopamine concentration series measurements were carried out in human blood plasma in 0.05 μM to 100 μM concentration range. Each concentration was measured immediately upon electrode immersion with a single cycle CV using a scan rate of 100 mV/s. Between successive concentration measurements, the electrodes were again kept immersed in blank 10 mM PBS.

## 3 Results and Discussion

The nanocellulosic materials and NC/MWCNT composite electrode architectures used in this work have been thoroughly characterized and reported elsewhere in previous works,^33,34^ and a summary of their different physical and chemical characteristics can be found in Supporting Information (Table S-1). In brief, the SCNF is composed of thread-like and highly sulfated (1.7 mmol/g of OSO^3–^ groups) fibrils that are ca. 4.2 ± 1.1 nm wide and 200–1000 nm long. The SCNC contains mostly crystalline rigid, rodlike structures with 0.17 mmol/g of OSO^3–^ groups, which are ca. 5.1 ± 1.7 nm wide and 100–200 nm long. Hydration properties of these two different NC grades and their specific interactions with proteins in different fouling environments are studied in this work with QCM-D, using single-component ultrathin films, and are discussed in the following sections.

### 3.1 Hydration of Nanocellulose

**Figure 1:**
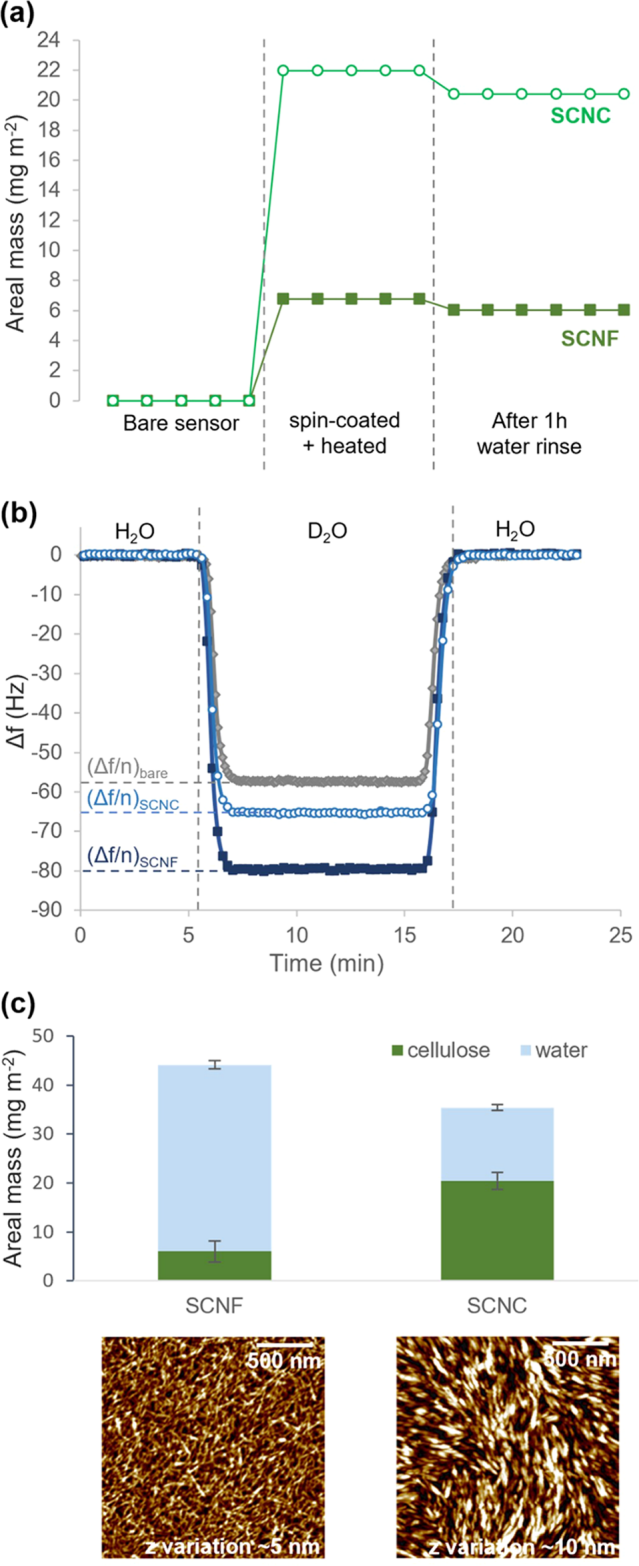
(a) Changes in areal mass at solid–air interface at different stages of deposition of SCNC and SCNF thin films as calculated based on changes in resonance frequency using the Sauerbrey equation. (b) Representative H_2_O/D_2_O solvent exchange data (fifth overtone) for bare and NC-coated QCM-D sensors, with horizontal dashed lines at the left edge labeled with the variables used in eq 1 to calculate the amount of water bound by the film Γ_water_ with eq 2. (c) Hydration of SCNF and SCNC thin films quantified based on dry areal mass and solvent exchange data, with corresponding AFM 2 × 2 μm^2^ height images of the films after swelling and subsequent drying.

Hygroscopicity and swelling tendencies of the different nanocellulosic components were investigated by following the hydration of SCNF and SCNC single-component ultrathin films with H_2_O/D_2_O solvent exchange in QCM-D, as presented in Figure 1. The areal masses of spin coated and dried SCNF and SCNC films are 6.0 ± 2.1 and 20.4 ± 1.7 mg/m^2^, respectively (Figure 1a). Based on an ellipsometry study conducted by Niinivaara et al., a layer of 20.4 mg/m^2^ corresponds to ca. 5 layers of SCNCs.^52^ The clearly lower mass of the SCNF film is only slightly more than that of a fibrillar monolayer. This relatively low mass of an ultrathin film is characteristic for low gelling point materials, as has been also found for highly charged cellulose nanofibers oxidized by 2,2,6,6-tetramethylpiperidine-1-oxyl (TEMPO) radical.^53^ Solvent exchange profiles in Figure 1b display instant leveling off of *Δf* after solvent exchange in all substrates (bare sensor, SCNC, and SCNF), indicating good accessibility of water in the hydrated films. Slower exchange kinetics with >20 min equilibration times have been previously observed for highly hydrated layers of carboxymethyl cellulose (CMC).^54^

Water content of the swollen SCNC film immersed in water is 42 ± 3 wt %, whereas the value for SCNF film is as high as 87 ± 4 wt % (Figure 1c). There is a vast variance of the corresponding values for SCNC reported in literature ranging from 21 wt %^55^ to 74 wt %.^49^ Our value fits well with the 37–48 wt % value reported by Niinivaara et al.^52^ for water vapor uptake at 97% RH where complete hydration is expectedly reached. Water binding of the fibrillar film is typically on a higher level than the crystalline film, already due to less dense fibrillar material packing, resulting in more space for free water. For instance, the value for native unoxidized CNF film was 71 wt % in a previous study.^54^ Furthermore, the network of fibrils is more flexible than that of CNCs, facilitating water uptake and thereby swelling of the film. Presence of highly charged ionic groups further increases the swelling tendency of the film, as osmotic pressure drives water into the layer to dilute the overall charge distribution in the system. The high hydration value obtained here for our SCNF film fits in the same range as 90 wt % value reported for highly charged (0.836 mmol/g) hemicellulose-containing TEMPO-oxidized CNF at 97% RH.^53^

### 3.2 Interactions with Protein Biofoulants

**Figure 2:**
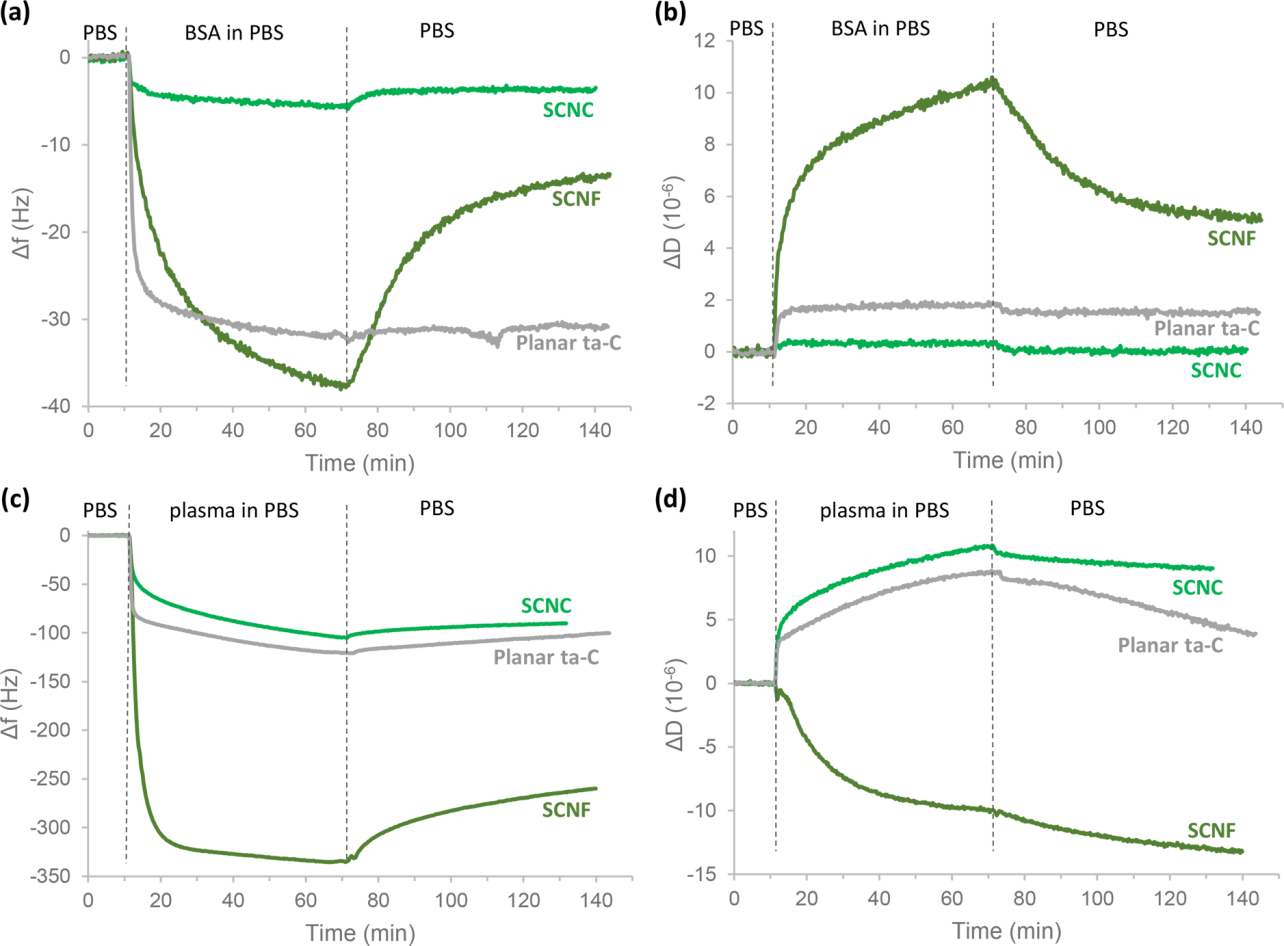
Changes in (a,c) frequency and (b,d) dissipation as a function of time for the adsorption of (a,b) 1 g/L BSA and (c,d) diluted human plasma (1:39) proteins from 10 mM PBS solution, with pH 7.4 at 25 °C on SCNF, SCNC, and planar ta-C. Note the difference in *y*-axis scales.

QCM-D data for the adsorption of BSA and diluted human plasma on SCNC, SCNF, and planar ta-C model surfaces are presented in Figure 2. On the ta-C surface, a moderately hydrated BSA layer is instantly formed and irreversibly attached (Figure 2a,b). Similar adsorption profile and adsorbed amount range has been recorded with QCM (*Δf* between −20 and −40 Hz) for adsorption of BSA from aqueous solution on other hydrophobic or polymeric surfaces (CH_3_-modified gold,^56^ polyester,^57^ polystyrene^58^). Adsorption of BSA on the SCNC surface is only minor, as indicated by the low values of *Δf* and *ΔD* (Figure 2a,b). Clearly higher adsorbed mass on SCNC has been reported by Aguilar-Sanchez et al.^59^ likely due to the >15 times higher ionic strength of medium used in their work. The highly hydrated SCNF surface results in a more substantial *Δf* over its crystalline counterpart, meaning a clearly higher wet mass is adsorbed from BSA solution (Figure 2a). The enrichment of the material is slow and largely reversible, as indicated by observed desorption during rinsing. The high *ΔD* of >5 × 10^–6^ indicates formation of a highly hydrated BSA layer (Figure 2b). This differs radically from the typical antifouling behavior characteristic for cellulosic materials such as regenerated cellulose, TEMPO-CNF, and CNF, where typically observed *Δf* reaches a maximum of −5 Hz and *ΔD* is ∼0.^54,60^

Weak affinity between the highly negatively charged SCNF and negatively charged BSA is expected to result from low amounts of positively charged carbamide groups (-CO-NH_2_) in the SCNF structure, created as a result of a side reaction during the sulfation process.^47^ This is indicated by elemental analysis which shows a slight excess amount of detected N compared to the amount of S (S 1.70 mmol/g vs N 1.97 mmol/g).^33^ Presence of N amount around 1.70 mmol/g can be explained by the chemical structure where an equal number of OSO_3_^–^ groups and their NH_3_^+^ counterions are present, while the excess 0.27 mmol/g is here suggested to result from carbamide groups. Nevertheless, the gel-like SCNF network appears to have excessive negative overall charge despite the occasional cationic spots, enabling the slow permeation and low, largely reversible enrichment of the hydrated BSA within the structure seen here. The high Δ*D*/−Δ*f* value of 0.275 Hz^–1^ for BSA adsorbed on SCNF (Table 1) indicates a very soft and loosely hydrated layer. This value is of the same order as those reported for the hydrated layers of anionic CMC adsorbed on cellulose in coiled conformation,^61^ where the water content can be as high as 97%.^62^

**Table 1:** Layer Thickness Based on Modeling of the QCM-D Data and Δ*D*/−Δ*f* (*n* = 5) after 1 h of Adsorption for BSA and Diluted Plasma on ta-C, SCNC, and SCNF^a^

	BSA		Diluted plasma	
	Layer thickness (nm)	Δ*D*/−Δ*f* (10^–3^ Hz^–1^)	Layer thickness (nm)	Δ*D*/−Δ*f* (10^–3^ Hz^–1^)
ta-C	7.0	58	27.8	71
SCNC	0.9	57	35.3	102
SCNF	11.5	275	–	–30

a Human plasma causes a much more drastic effect than BSA on any of the used surfaces, including adsorption of heavy mass as well as structural changes in the original hydrated NC films.

Human plasma, consisting of a variety of proteins and ions, shows a dramatically stronger influence on the surfaces than the BSA protein alone (Figure 2c,d). Instant adsorption of a relatively heavy hydrated layer is observed on ta-C, with mass and hydration further increasing with time, the layer thickness reaching ∼28 nm after 1 h of adsorption (Table 1). The layer formed on SCNC develops slightly more slowly and is more hydrated than that on the rather hydrophobic ta-C, indicating weaker affinity and less conformational rearrangements of plasma components toward SCNC. In the case of SCNF, drastic simultaneous decreases in both *Δf* and *ΔD* indicate a massive increase of wet mass simultaneously with substantial dehydration and densifying of the originally hydrated SCNF film. Due to the complete transformation of the original substrate because of dehydration during adsorption, it is not possible to extract detailed information on the thickness or hydration level of the actual adsorbing protein layer based on QCM-D data. The reason for this strong dehydration of SCNF is expected to be charge neutralization by the multivalent cationic components of the plasma as well as accumulation of protein components, disturbing the coordination of bound water and decreasing the volume for free water within the film.

### 3.3 Electroanalytical Performance in Different Fouling Environments

**Figure 3:**
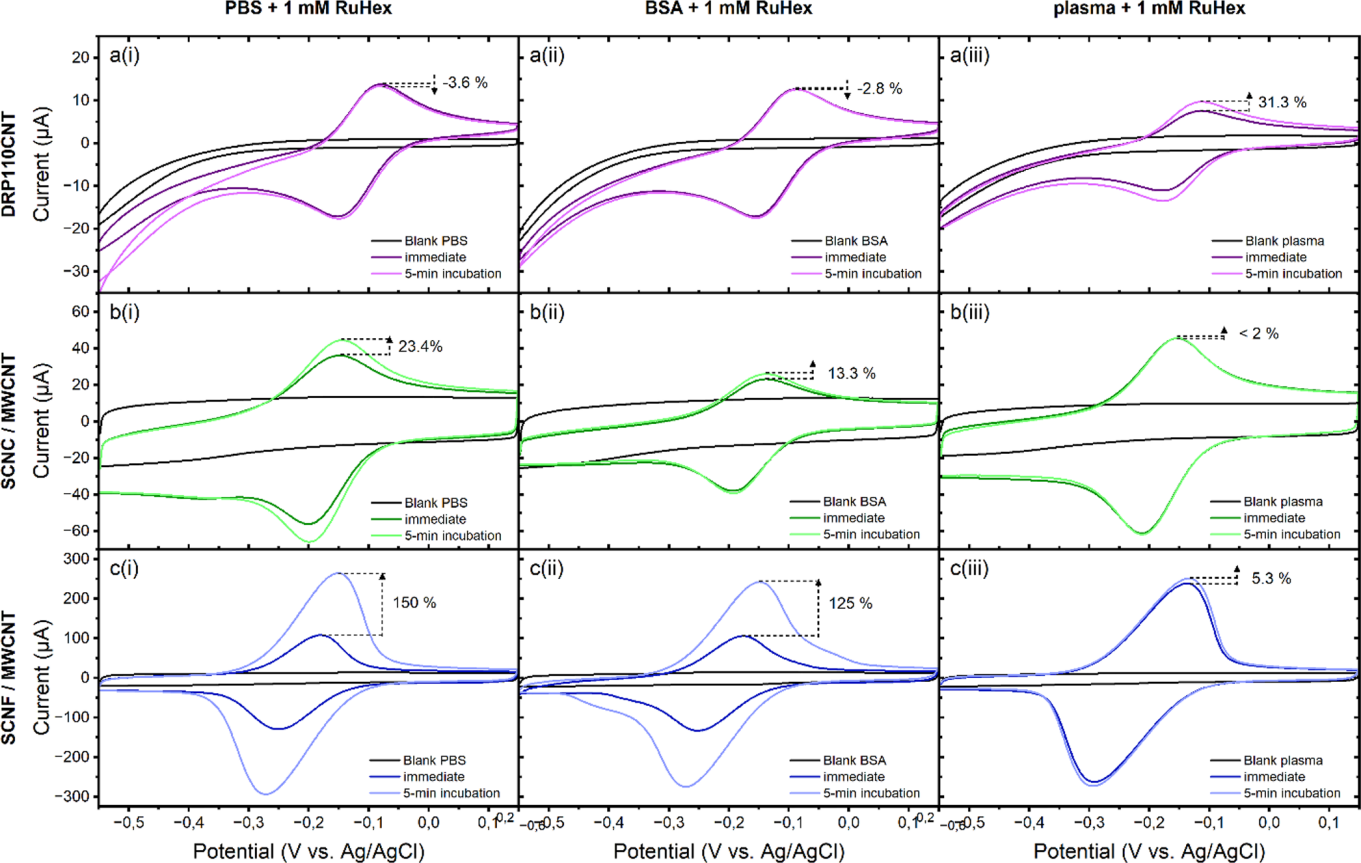
Electrochemical response toward the OSR probe RuHex in PBS (i), 4 wt % BSA in PBS (ii), and human plasma (iii) for commercial DRP110CNT electrode (a) compared to SCNC/MWCNT (b) and SCNF/MWCNT (c) composite electrodes. The increase/decrease in peak oxidation currents over the 5 min incubation are also indicated in each graph.

The effects of protein adsorption on the electroanalytical behavior of the NC/MWCNT composite electrodes and commercial MWCNT electrode without NC were studied over a 5 min incubation period, in the different measurement environments. This time frame has been chosen to represent a reasonable period of the measurement window in a point-of-care medical sensor application. Further, the QCM-D studies indicate that the protein adsorptions start almost instantaneously at all studied surfaces, making it reasonable to expect that any fouling effects can also be observed within the 5 min incubation measurement window. CV measurements of 1 mM RuHex at 100 mV/s scan rate using the different electrodes, obtained immediately at the point of their immersion and after 5 min wait in the measurement electrolytes (PBS, BSA or human plasma), are shown in Figure 3, along with the corresponding background currents measured in the blank electrolytes.

The redox currents observed for 1 mM RuHex were significantly higher at the NC/MWCNT composites compared to the commercial MWCNT electrodes (note the progressively increasing *y*-axis scale in Figure 3a–c). The SCNF/MWCNT (Figure 3c) composites showed over 10-fold increase in redox currents compared to the commercial MWCNT electrodes. In PBS, both the NC/MWCNT electrodes showed an increase in redox current over the 5 min incubation period, whereas no such enrichment was observed in the commercial MWCNT electrodes without NC. A similar trend was also observed at all electrode types in the 4 wt % BSA solution, but with slightly lower redox currents compared to that observed in PBS. The greatest difference was observed at the SCNC/MWCNT electrode, where the RuHex oxidation current was on average 19% less in the BSA solution (Figure 3b(ii)) than that in PBS (Figure 3b(i)), after the 5 min incubation. All studied electrode types behaved drastically different in human plasma, compared to either PBS or 4 wt % BSA systems. Interestingly, both the NC/MWCNT composites showed much higher immediate currents for RuHex in human plasma (Figure 3b(iii),c(iii)), comparable to the currents observed only after 5 min incubation in PBS or BSA. Additionally, no significant change in current was observed at either of these electrodes after 5 min incubation in human plasma. In contrast, the commercial MWCNT electrode without NC showed a lower immediate current compared to its response in PBS or 4 wt % BSA, and a slight increase in redox currents was observed after 5 min of incubation of this electrode type in human plasma.

**Figure 4:**
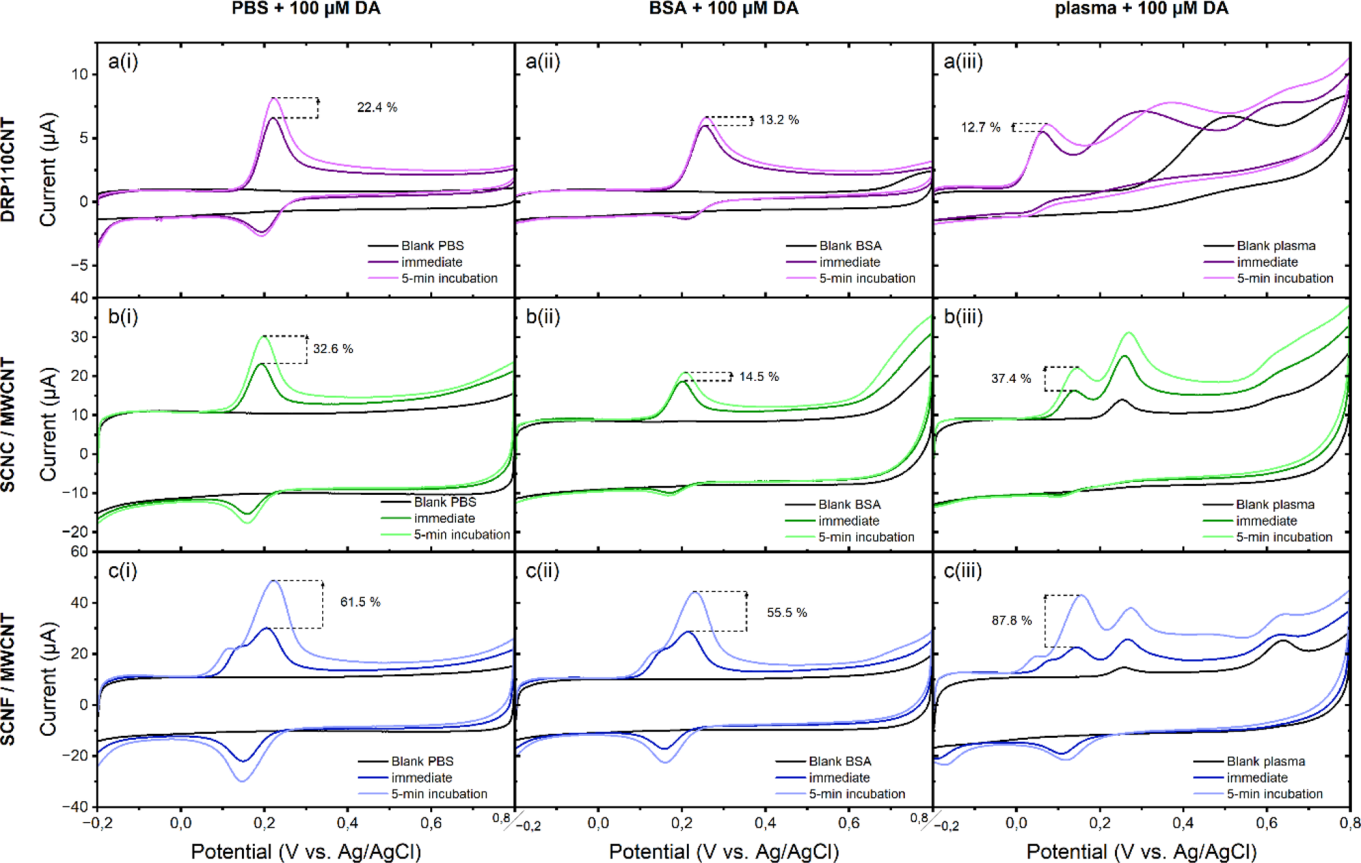
Electrochemical response toward ISR probe DA in PBS (i), 4 wt % BSA in PBS (ii), and human plasma (iii) for commercial DRP110CNT electrode (a) compared to SCNC/MWCNT (b) and SCNF/MWCNT (c) composite electrodes. The increase/decrease in peak oxidation currents over the 5 min incubation are also indicated in each graph.

Electrode behaviors toward more complex inner sphere redox molecules in the different fouling environments were evaluated using a 100 μM DA concentration. Immediate and 5 min incubation CV measurements are shown in Figure 4, along with the background currents in the −0.2 to +0.8 V potential range. Overall, the redox currents for DA are seen to be clearly higher at the NC/MWCNT composites in all electrolytes compared to the commercial MWCNT without NC, as was also seen with the OSR probe RuHex response. However, DA has been suggested to bind spontaneously with BSA via hydrogen bonding and van der Waals forces.^63,64^ Dopamine binding to plasma proteins has been reported by Franksson and Änggård^65^ to reach ca. 13% at physiologically relevant concentrations (<10^–6^ M) and ca. 8% at higher concentrations. This binding could partially explain the lower redox currents for DA, to an order of ca. 10–25%, observed here in the presence of 4 wt % BSA and human plasma, compared to pure PBS.

Additionally, the DA molecule has also been shown to cause electrochemical fouling due to the formation of polydopamine,^66−68^ thereby resulting in loss of electrode stability over prolonged measurements. The DA redox mechanisms involve adsorption onto the electrode surface and are therefore highly sensitive to the specific surface chemistry of the electrode materials. Redox responses of the commercial MWCNT electrode and SCNC/MWCNT composite electrode (Figure 4a,b) show a similar trend in PBS and BSA, with primary oxidation peak onset potential around 0.17 V in both cases. The redox currents are lower in BSA for both electrode types, compared to PBS, with the SCNC/MWCNT composite showing a stronger decrease compared to the commercial MWCNT. In contrast, the SCNF/MWCNT composite shows a significantly different redox response toward DA, with a strong oxidation prepeak onset around 0.16 V in both PBS and BSA (Figure 4c(i),(ii)). This oxidation prepeak evolution is an indication of oxidation product adsorption on an electrode surface.^34^ As has been reported in previous detailed characterizations of the composites,^33,34^ the surface chemistry of MWCNT remains unaltered in the composites with different nanocellulosic materials. Thus, the clear difference observed in DA redox response between the SCNF/MWCNT and SCNC/MWCNT composites can primarily be attributed to morphological differences between the composites, resulting from the nanocellulosic components. The more hygroscopic and porous SCNF/MWCNT composite appears to enable the access to specific MWCNT surface sites that are favorable for the adsorption of the DA oxidation product, dopamine quinone (DAQ), resulting in a clear oxidation prepeak and consequently a more intense reduction peak for DAQ to DA in the CV. SCNC/MWCNT composite and DRP110CNT electrodes do not appear to adsorb DAQ, allowing DAQ left in the solution to go through a chemical reaction into dopamine chrome, diminishing the intensity of DAQ reduction back to DA. This strong DAQ adsorption on SCNF/MWCNT composites may also be a cause for slower DA enrichment in plasma than what was observed for RuHex, where immediate measurement displayed similar redox current as after incubation due to rapid wetting. DAQ adsorption may affect SCNF-based networks in a way that it maintains a more open architecture even in plasma, an indication of this is the formation of gels between DA and hyaluronic acid,^69^ which could be why similar behavior was not seen in ISR measurements in plasma as was seen in OSR measurements.

Further, the behavior of all three electrode types in human plasma at the −0.2 to +0.8 V potential range (ISR potential range) is considerably different from the behavior observed at the −0.55 to +0.15 V potential range (OSR potential range) used to study the RuHex probe and requires careful evaluation. For instance, the background CV measurements in human plasma (black curves in Figure 4a(iii),b(iii),c(iii)) in the ISR potential range clearly show two oxidation peaks, the first between 0.2 and 0.4 V, and another between 0.6 and 0.8 V, respectively. The shape and intensity of these two background related peaks are also clearly different among the three different electrode types. The first oxidation peak, occurring as a broad peak at around 0.4 V at the commercial MWCNT electrode, is observed to be sharper and cathodically shifted to around 0.3 V at the NC/MWCNT composite electrodes. This peak can be attributed to the oxidation of uric acid, which is often present in human plasma in the concentration range of 160–470 μM.^70,71^ The intensity of this uric acid oxidation peak is seen to be clearly smaller at the NC/MWCNT composites, compared to the commercial MWCNT, and can be explained by the predominantly negatively charged nanocellulosic materials in the composites having a repulsive effect toward the anionic uric acid molecules. The second, broader oxidation peak observed at around 0.7 V could be attributed to the oxidation of multiple components. For example, amino acids such as tyrosine or tryptophan are typically present in the 30–50 μM concentration range in human plasma and typically oxidize at ca. 0.7–1.0 V.^72,73^ Additionally, vitamins A and B as well as molecules such as xanthine have been reported to oxidize around this potential range and can play a part in the intensity of the oxidation peak.^74,75^

Despite the high background interference in human plasma from various competing molecules, all three studied electrode types can clearly detect 100 μM DA. The primary DA oxidation peak exhibits a slight cathodic shift at all electrode types, with the commercial MWCNT showing the highest shift. This shift is likely due to the competitive adsorption of other molecules, such as cations and other ions present in human plasma, as discussed earlier. Similar to the behavior in PBS and BSA, the commercial MWCNT electrode shows only a small increase in redox currents for 100 μM DA and plasma interferents after the 5 min incubation. Simultaneously, there appears to be a slight anodic shift of the oxidation peaks and tilt in the CV baseline, indicating fouling of the electrode surface. On the other hand, the NC/MWCNT composites show a more pronounced increase in the DA and interferent oxidation currents, with no noticeable shift in oxidation potentials. Moreover, at the SCNF/MWCNT composite, the DA oxidation peak current appears to be selectively more enriched with the 5 min incubation, compared to the anionic uric acid oxidation peak. This further supports the hypothesis that the highly negatively functionalized SCNF/MWCNT composite enables ion selective enrichment of analytes, resulting in improved sensitivity and selectivity.

The behaviors of RuHex and DA at the composite electrodes were also evaluated using scan rate series CV measurements from 25 to 500 mV/s scan rates. The slope of linear fits of the log of oxidation peak currents versus log of scan rates were estimated for both RuHex and DA in PBS, BSA and plasma environments (Supporting Information Figure S-4a,b, respectively). These slopes provide useful insight into the electrochemical processes occurring at the electrodes. A theoretical slope of 0.5 indicates a diffusion (linear semi-infinite) controlled process, and a slope closer to 1 indicates the involvement of adsorption processes. The estimated slopes for SCNF/MWCNT composite were 0.6, 0.86 and 0.87, and for SCNC/MWCNT composites 0.75, 0.81 and 0.76, respectively, in PBS, BSA and plasma. This indicates that the solution diffusion dominates RuHex behavior at the SCNF/MWCNT electrode in PBS, and in all other cases, surface adsorption processes play a dominant role. For DA, the estimated slopes for SCNF/MWCNT composite were 0.74, 1.27 and 0.88, and for SCNC/MWCNT composites 0.87, 0.97 and 0.75, respectively, in PBS, BSA and plasma. This indicates that the oxidation of DA at both SCNF/MWCNT and SCNC/MWCNT composite electrodes in all studied environments is primarily controlled by surface adsorption processes as discussed earlier. These results further indicate that the protein adsorption from different fouling environments does not drastically alter the analyte diffusion or adsorption mechanisms within the NC/MWCNT composite electrodes.

### 3.4 Dopamine Concentration Measurements in Human Plasma

**Figure 5:**
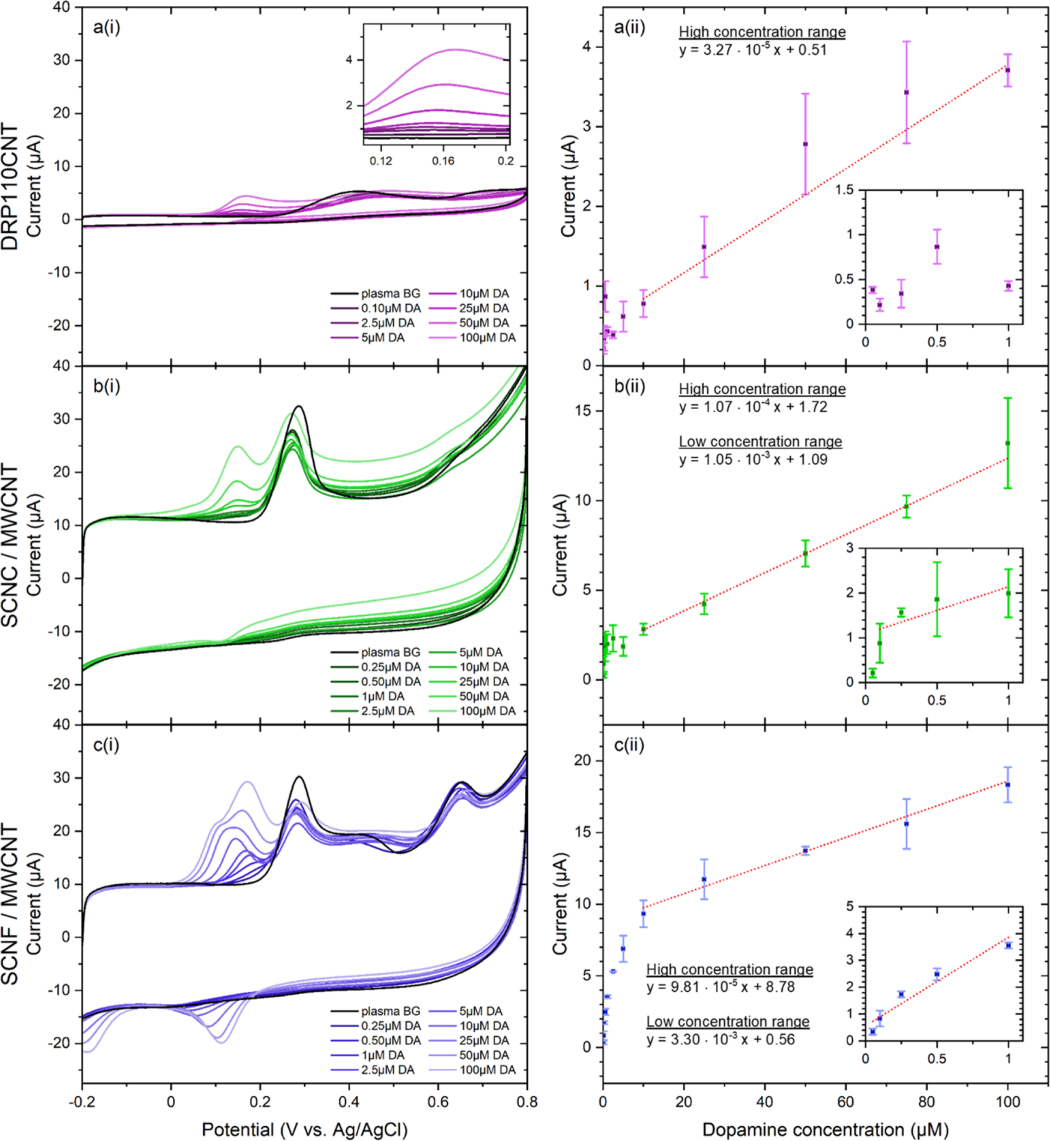
Dopamine concentration series and corresponding current calibration curves, measured immediately upon immersion, in human plasma at 100 mV/s scan rate, for commercial DRP110CNT (a(i), a(ii)), SCNC/MWCNT (b(i), b(ii)), and SCNF/MWCNT (c(i), c(ii)) composite electrodes.

The advantages of NC/MWCNT composite electrodes and their applicability to medical sensor development were further evaluated by measuring a current-concentration series for DA in human plasma, starting from a physiologically relevant concentration of 50 nM and up to 100 μM. Consecutive concentrations were achieved by adding necessary amount of DA stock solution to the electrochemical cell containing human plasma. CV measurements for all three electrode types, measured immediately upon electrode immersion in each concentration solution, are shown in Figure 5, along with the corresponding current vs concentration calibration curves. Limit of detection (LOD) was calculated using the equation 3.3 × σ/*S*, where the constant 3.3 represents 5% probabilities for false positive and false negative values, σ is the standard deviation of background current at the DA oxidation potential measured in absence of DA (*n* = 3), and *S* is the electrode sensitivity (μA/nM) toward DA, obtained as the slope of the linear fit in the current vs concentration plot.^76^

Sensitivity and LOD of each electrode type are shown in Table 2, separately for the physiologically relevant concentration range (0.05–1 μM) and high concentration range (10–100 μM). As seen in Figure 5a(i), the commercial MWCNT electrode does not produce a clear discernible oxidation peak for DA at concentrations below 10 μM, and therefore, the estimation of DA oxidation current at these low physiologically relevant concentrations is unreliable. The LOD value for DA at these electrodes can therefore only be reliably estimated at concentrations above 10 μM. A similar trend is also observed at the SCNC/MWCNT electrode, where a clear DA oxidation peak is observable only above 10 μM concentration. At lower concentrations, the DA oxidation can only be observed as a broad increase in background current at the expected potential, thereby making the LOD estimation at low concentrations unreliable (*R*^2^ for linear fit is very low) also at the SCNC/MWCNT electrodes.

**Table 2:** SCNF/MWCNT, SCNC/MWCNT and DRP110CNT Sensitivity, Coefficient of Determination, and LOD for Physiologically Relevant DA Concentration Range (0.05–1 μM) and High DA Concentration Range (10–100 μM)

	Physiologically relevant range			High concentration range		
Composite	Sensitivity (μA/nM)	*R*^2^	LOD (nM)	Sensitivity (μA/nM)	*R*^2^	LOD (nM)
SCNF/MWCNT	3.30 × 10^–3^	0.939	21.6	9.81 × 10^–5^	0.987	727
SCNC/MWCNT	1.05 × 10^–3^	0.695	51.3	1.07 × 10^–4^	0.998	506
DRP110CNT	–	–	–	3.27 × 10^–5^	0.985	218

In contrast, the SCNF/MWCNT composite shows a clear shoulder for DA oxidation already for 0.5 μM, with strong peak evolving at around 0.17 V, for 1 μM DA concentration (Figure 5c(i)). At low analyte concentrations, the DA adsorption effects are more predominant and the observed clear oxidation peaks for DA at the SCNF/MWCNT electrode are likely due to the favorable product adsorption sites in this composite. With higher DA concentrations, the oxidation peak at SCNF/MWCNT composite appears to broaden due to higher analyte diffusion, and the adsorption related peak is shifted cathodically, resulting in the evolution of the prepeak, clearly observed at DA concentrations above 50 μM. The differences in adsorption and diffusion mechanisms result in two distinct linear regions for DA detection at the SCNF/MWCNT composite electrodes. At lower DA concentrations (0.05–1 μM), a steeper slope is obtained, resulting in a LOD value of 21.6 nM, whereas at higher concentrations, prepeak evolution causes a slower increase in main oxidation peak, reducing the slope and therefore the LOD to 727 nM.

To summarize, the electrochemical measurements from 5 min incubation in fouling environments, as well as DA concentration series in human plasma, clearly demonstrate the superior performance of NC/MWCNT composites compared to commercial MWCNT without NC. Both QCM-D and electrochemical measurements indicate that the adsorption of proteins from human plasma is significantly different from that of just BSA adsorption. QCM-D results indicate that the BSA forms a thin, moderately hydrated layer on both amorphous carbon and SCNC, compared to plasma proteins which form thicker but looser and more hydrated layers. Correspondingly, the RuHex measurements (Figure 3) indicate that the pure BSA adsorption results in stronger passivation effects at all electrodes compared to human plasma proteins. Interestingly, the commercial MWCNT electrode appears to be slightly more resistant to BSA adsorption, with higher recoveries for both RuHex and DA, compared to the SCNC/MWCNT composite. From previous studies,^34^ we know that the SCNC/MWCNT composite has a dense film architecture resulting from rigid cellulose nanocrystals, aligned closely around MWCNT. Together with the relatively low hydration of the SCNC and the tendency for BSA adsorption seen in QCM-D, the electrochemical results also indicate that the SCNC/MWCNT composite does not provide the optimal electroanalytical performance in BSA. On the contrary, although QCM-D results indicate similar adsorption trends for human plasma on both amorphous carbon and SCNC surfaces, the electrochemical results indicate that the SCNC/MWCNT composite offers better performance in human plasma compared to commercial MWCNT electrodes, for both OSR and ISR analytes. Especially in the case of DA, a clear improvement in both selectivity and sensitivity is observed at the SCNC/MWCNT composite due to the better wetting and ionic conductivity provided by the NC component.

The SCNF/MWCNT composite on the other hand shows drastically different and improved electroanalytical performance at all studied systems, for both the OSR and ISR analytes. Although significant BSA adsorption is observed at the SCNF surface in QCM-D studies, the adsorbed layer appears to be more hydrated and partially reversible. Correspondingly, the electrochemical results show that the SCNF/MWCNT composite behavior in BSA is quite similar to that in PBS. On the other hand, QCM-D results for human plasma protein adsorption at SCNF surface revealed an interesting and surprising behavior, indicative of a rapidly forming heavy adsorbed layer that drastically alters the hydration and density of the original SCNF model film. Further investigations are required to extract specific information about the affinities and interaction mechanisms of various components of complex plasma toward SCNF during the fouling process. However, the SCNF/MWCNT composites clearly outperform other electrode types in the electrochemical studies, especially in human plasma. The porous SCNF/MWCNT architecture^34^ together with very high degree of hydration of SCNF appears to make this electrode system more resistant to passivation in both BSA and human plasma. Additionally, the high degree of negative functional group substitution clearly improves ionic selectivity and sensitivity, as seen in the DA enrichment and concentration series studies in human plasma.

## 4 Conclusions

Our study focuses on the outstanding electroanalytical properties of NC/MWCNT composite membranes and their potential for sensor applications in complex biological matrices containing proteins. The unique wetting behavior of the NC containing composite materials provides significant advantages for small molecules monitoring in biological environments that cannot be achieved with purely carbon- or nanocarbon-based approaches. The nanocellulosic material properties are shown to have a significant effect on the hydration and protein adsorption mechanisms in different electrolyte solutions. Consequently, the electroanalytical performance of their composites with MWCNT is also strongly influenced by the choice of nanocellulosic materials. The highly negatively functionalized SCNF appears to result in a more viable matrix material for use in sensitive electrochemical monitoring due to its better wetting properties. In contrast, the crystalline SCNC results in a less optimal, denser membrane architecture when combined with MWCNTs, and due to its lesser hydration tendency, it does not improve the electroanalytical performance of the composite membrane as much as the presence of SCNF does. Our research further highlights the importance of considering the material choices and their interactions with complex test matrices closer to actual measurement environments in biosensor development. The presence of human plasma, for example, is shown to drastically alter the protein fouling layer adsorption mechanisms at different carbon and cellulosic surfaces and, correspondingly, the electroanalytical performance of their composites, compared to just a simple BSA model system. To summarize, our results clearly demonstrate that the inclusion of nanocellulosic materials in composites with MWCNT offers much superior electroanalytical performance compared to commercial MWCNT electrodes without NC in terms of both sensitivity and electrode fouling. Together with a careful choice of nanocellulosic material properties, such composite material platforms have the potential to revolutionize point-of-care sensor development for healthcare technology, where fast and consistent results are crucial.

## Supporting Information Available

Detailed protocol for QCM-D sample preparation; chemical and physical properties of NC and their composites with MWCNTs: TEM images; UV–vis spectra of composite dispersions and CV of corresponding composite films, freshly prepared and after two years. OSR- and ISR-probe redox reaction characteristics of the composite films: redox-peak current difference and peak separation for ruthenium hexamine and dopamine; effect of scan rates on the oxidation currents for ruthenium hexamine and dopamine in different environments (PDF)
