# Supplementary material for: Protein Adsorption and Its Effects on Electroanalytical Performance of Nanocellulose/Carbon Nanotube Composite Electrodes
Source: Biomacromolecules. 2023 Jul 11;24(8):3806–18. doi: 10.1021/acs.biomac.3c00449 (PMC10428158; doi:10.1021/acs.biomac.3c00449)
Supplement: Supplementary file 1 — bm3c00449_si_001.pdf [file bm3c00449_si_001.pdf]

Supporting information

Protein Adsorption and Its Effects on Electroanalytical Performance of Nanocellulose / Carbon Nanotube Composite Electrodes

Touko Liljeström,[a] *Katri S. Kontturi,*[b*] *Vasuki Durairaj,*[a,b*] *Niklas Wester,*[a, c] *Tekla Tammelin,*[b] *Tomi Laurila,*[a,c] *and Jari Koskinen.*[a]

[a] Department of Chemistry and Materials Science, School of Chemical Technology, Aalto University, P.O. Box 16100, 00076 Aalto, Finland.

[b] VTT Technical Research Centre of Finland, Sustainable Products and Materials, P.O. Box 1000, FI-02044 VTT, Finland.

[c] Department of Electrical Engineering and Automation, School of Electrical Engineering, Aalto University, PO Box 13500, 00076 Aalto, Finland.

Detailed QCM-D protocol for sample preparation

A 7 nm thick ta-C layer was deposited on silica-coated QCM-D sensors by pulsed filtered cathodic vacuum arc (p-FCVA), utilizing an underlying sputtered 20 nm thick Ti adhesive layer. Ultrathin films of SCNF and SCNC were deposited by spin coating (WS-650SX-6NPP/LITE spin coater, Laurell Technologies Corp., North Wales, PA, USA) on AT cut gold-covered QCM-D sensor with fundamental resonance frequency $f_0 \approx 5$ MHz (Biolin Scientific, Gothenburg, Sweden). SCNC films were spin-coated from 10 g l:1 aqueous suspension of SCNC freshly stirred with ultrasonic microtip for 2 mins with 25% amplitude to ensure homogeneity throughout the suspension. Respectively for SCNF films, 2 g l:1 aqueous suspension of SCNF was ultrasonicated for 10 mins with 25% amplitude in order to disintegrate possible SCNF aggregates, and then centrifuged at 10 400 rpm for 40 mins. The supernatant of the centrifuged solution, containing individual nanofibers, was used for spin coating. Spin coating of both SCNF and SCNC suspensions was conducted at 3000 rpm for 1 min. A thin layer of polyethyleneimine (PEI) was used as an anchoring polymer, adsorbed on the bare substrate freshly cleaned in a UV/ozonator (Bioforce Nanosciences, Ames, Iowa). After spin coating, the SCNC and SCNF films were annealed at 80 °C for 15 mins and 30 mins, respectively, after which they were immersed in water for 1 hour to remove any unattached material, and subsequently dried.

Chemical and physical properties of nanocellulose and their composites

Previously established physicochemical properties of the used nanocellulose materials as well as composite film thicknesses are shown in table S-1 [18,31]. SCNF / MWCNT membranes were observed to form as thicker and more open films with higher charge density than SCNC / MWCNT membranes.

Table S-1. Summary of physicochemical properties of the functionalized nanocellulosic materials and drop-cast NC/MWCNT membranes [31].

nanocellulose	SCNF	SCNC
charge density (mmol/g)	1.7 $[OSO_3^-]$	0.17 $[OSO_3^-]$
ζ-potential (mV)	-35.7 ± 0.5	-35 ± 1.4
width (mm)	4.2 ± 1.1	5.1 ± 1.7
**composite material**	**SCNF/MWCNT**	**SCNC/MWCNT**
thickness (µm)	2.27 ± 0.4	1.82 ± 0.1

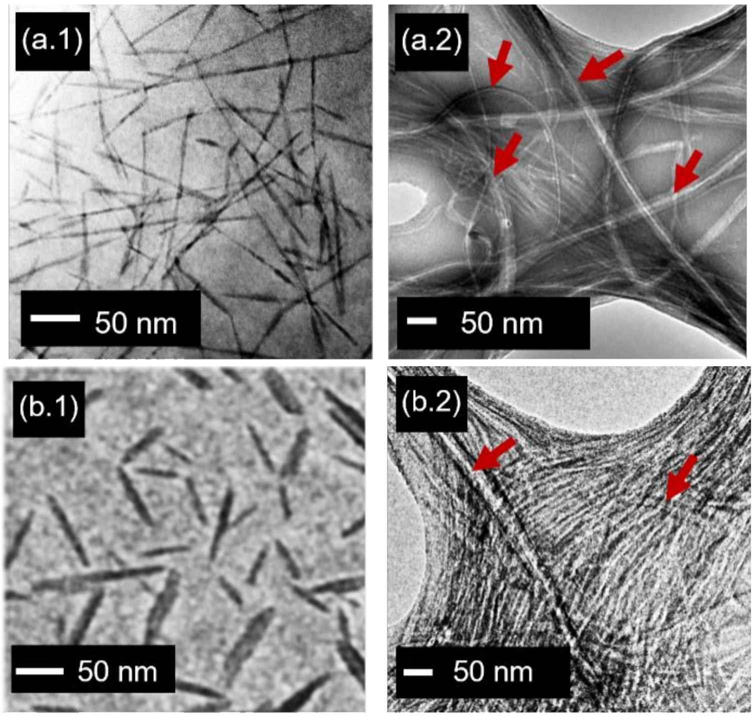

Figure S-1. TEM images of the SCNF (a.1), SCNF / MWCNT composite (a.2), SCNC (b.1) and SCNC / MWCNT composite (b.2) materials.

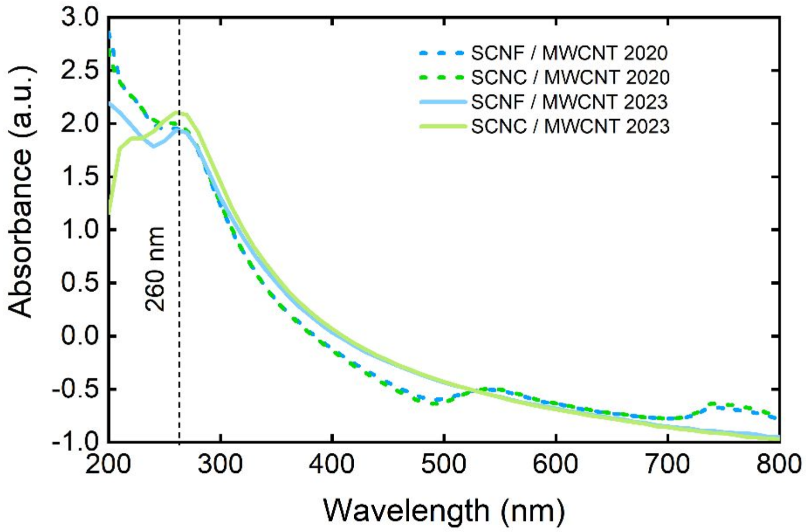

Figure S-2. UV-visible absorption spectra for SCNF / MWCNT and SCNC / MWCNT composite suspensions – showing the normalized absorbance spectra for the freshly made 1000x diluted samples measured in 2020 and the same samples remeasured in 2023. The absorbance peak at ~260 nm corresponding to debundled MWCNTs is seen clearly in all spectra.

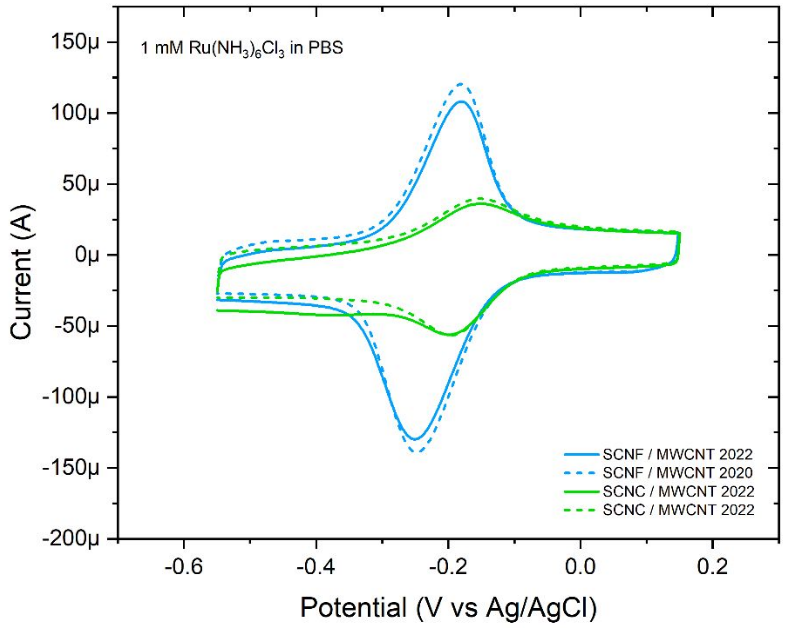

Figure S-3. Cyclic voltammetry measurement of 1 mM RuHex in PBS at electrodes made in 2020 and 2022, from same composite suspensions of SCNF / MWCNT and SCNC / MWCNT, indicating the stability of suspensions with respect to end applications.

OSR- and ISR-probe redox reaction characteristics

Oxidation peak potential (E_{ox}) and redox-peak separation (ΔE_p) are collected in table S-2, based on Figures 3 and 4 in the manuscript. SCNC / MWCNT electrodes consistently resulted in a more stable and closer to reversible ΔE_p towards the OSR probe regardless of measurement medium and enrichment time. A much more drastic change was observed for SCNF / MWCNT electrodes during the enrichment period, due to strong electrostatic and adsorption effects.

Table S-2. Redox-peak current difference (I_{pa}/I_{pc}) and redox-peak separation (ΔE_p) for SCNF / MWCNT and SCNC / MWCNT for 1 mM hexaammineruthenium (III) chloride and 100 µM dopamine in 10 mM PBS, 4 wt% BSA in PBS and human plasma.

	hexaammineruthenium (III) chloride				dopamine			
	SCNF/MWCNT		SCNC/MWCNT		SCNF/MWCNT		SCNC/MWCNT	
	I_{pa}/I_{pc}	ΔE_p (mV)	I_{pa}/I_{pc}	ΔE_p (mV)	I_{pa}/I_{pc}	ΔE_p (mV)	I_{pa}/I_{pc}	ΔE_p (mV)
PBS, immediate	-182	65.8	-152	47.9	204	51.9	190	28.0
PBS, enrich	-154	116	-148	50.0	222	73.9	196	36.0
BSA, immediate	-178	71.9	-140	52.0	212	51.9	198	23.9
BSA, enrich	-150	120	-140	51.9	230	67.9	206	38.0
plasma, immediate	-140	150	-158	53.9	142	32	138	34.0
plasma, enrich	-136	158	-156	55.9	154	33.9	142	35.9

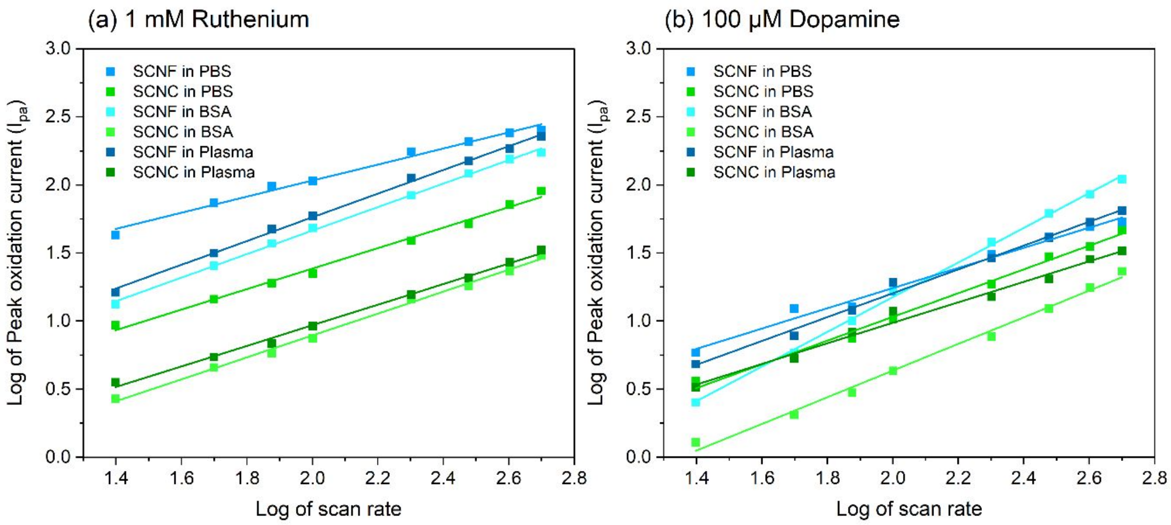

Ruthenium in PBS at SCNF / MWCNT	
Equation	y = 0.85237 + 0.59003*x
R-Square (COD)	0.98814
Slope	0.59003 ± 0.02639

Ruthenium in PBS at SCNC / MWCNT	
Equation	y = -0.11969 + 0.75281*x
R-Square (COD)	0.99248
Slope	0.75281 ± 0.02676

Ruthenium in BSA at SCNF / MWCNT	
Equation	y = -0.05964 + 0.86237*x
R-Square (COD)	0.99819
Slope	0.86237 ± 0.015

Ruthenium in BSA at SCNC / MWCNT	
Equation	y = -0.71643 + 0.80553*x
R-Square (COD)	0.99632
Slope	0.80553 ± 0.01998

Ruthenium in Plasma at SCNF / MWCNT	
Equation	y = 0.0213 + 0.87066*x
R-Square (COD)	0.99759
Slope	0.87066 ± 0.01748

Ruthenium in Plasma at SCNC / MWCNT	
Equation	y = -0.54272 + 0.7564*x
R-Square (COD)	0.99562
Slope	0.7564 ± 0.02048

Dopamine in PBS at SCNF / MWCNT	
Equation	y = -0.24013 + 0.74133*x
R-Square (COD)	0.98693
Slope	0.74133 ± 0.03483

Dopamine in PBS at SCNC / MWCNT	
Equation	y = -0.71336 + 0.87245*x
R-Square (COD)	0.99324
Slope	0.87245 ± 0.02938

Dopamine in BSA at SCNF / MWCNT	
Equation	y = -1.37174 + 1.27419*x
R-Square (COD)	0.99793
Slope	1.27419 ± 0.02368

Dopamine in BSA at SCNC / MWCNT	
Equation	y = -1.32212 + 0.97964*x
R-Square (COD)	0.99247
Slope	0.97964 ± 0.03485

Dopamine in Plasma at SCNF / MWCNT	
Equation	y = -0.54747 + 0.87599*x
R-Square (COD)	0.99207
Slope	0.87599 ± 0.03198

Dopamine in Plasma at SCNC / MWCNT	
Equation	y = -0.52068 + 0.75426*x
R-Square (COD)	0.98543
Slope	0.75426 ± 0.03744

Figure S-4. Effect of scan rates on the oxidation currents for 1 mM ruthenium hexamine (a) and 100 μM dopamine (b) in the different measurement environments (PBS, BSA and human plasma) for SCNF / MWCNT and SCNC / MWCNT composite electrodes. The current-scan rate relationship is represented by the linear fit of their logarithmic values, and the corresponding linear equations are also presented.
